# Genistein Sensitizes Human Cholangiocarcinoma Cell Lines to Be Susceptible to Natural Killer Cells

**DOI:** 10.3390/biology11081098

**Published:** 2022-07-23

**Authors:** Chutipa Chiawpanit, Suthida Panwong, Nunghathai Sawasdee, Pa-thai Yenchitsomanus, Aussara Panya

**Affiliations:** 1 Doctoral Program in Biology, Faculty of Science, Chiang Mai University, Chiang Mai 50200, Thailand; chutipa_c@cmu.ac.th; 2 Department of Biology, Faculty of Science, Chiang Mai University, Chiang Mai 50200, Thailand; suthida_panw@cmu.ac.th; 3 Doctoral Program in Applied Microbiology, Faculty of Science, Chiang Mai University, Chiang Mai 50200, Thailand; 4 Siriraj Center of Research Excellence for Cancer Immunotherapy (SiCORE-CIT), Research Department, Faculty of Medicine, Siriraj Hospital, Mahidol University, Bangkok 10700, Thailand; nunghathai.saw@mahidol.edu (N.S.); pa-thai.yen@mahidol.edu (P.-t.Y.); 5 Division of Molecular Medicine, Research Department, Faculty of Medicine, Siriraj Hospital, Mahidol University, Bangkok 10700, Thailand; 6 Research Center in Bioresources for Agriculture, Industry and Medicine, Faculty of Science, Chiang Mai University, Chiang Mai 50200, Thailand

**Keywords:** cholangiocarcinoma, genistein, sensitization, apoptosis, death receptors, NK-92 cells

## Abstract

**Simple Summary:**

Cholangiocarcinoma (CCA) is a malignancy of bile duct epithelial cells, which has a poor prognosis and high mortality. Consequently, effective therapeutic options are required to improve the treatment outcome. In the present study, we demonstrated the effects of genistein on sensitizing CCA and enhancing natural killer (NK-92) cells to induce cytotoxicity of CCA cells. Genistein treatment could promote extrinsic apoptotic pathway to induce CCA cell death. Mechanistically, genistein treatment decreased the expression of anti-apoptotic protein, cFLIP, and significantly upregulated the expression of death receptors (Fas receptor; FasR, and TRAIL receptor; TRAIL-R), which might contribute to the increased susceptibility of CCA to NK-92 cells.

**Abstract:**

Cholangiocarcinoma (CCA) is a lethal bile duct cancer, which has poor treatment outcomes due to its high resistance to chemotherapy and cancer recurrence. Activation of aberrant anti-apoptotic signaling pathway has been reported to be a mechanism of chemoresistance and immune escape of CCA. Therefore, reversal of anti-apoptotic signaling pathway represents a feasible approach to potentiate effective treatments, especially for CCA with high chemoresistance. In this study, we demonstrated the effects of genistein on reactivation of apoptosis cascade and increase the susceptibility of CCA cells to natural killer (NK-92) cells. Genistein at 50 and 100 µM significantly activated extrinsic apoptotic pathway in CCA cells (KKU055, KKU100, and KKU213A), which was evident by reduction of procaspase-8 and -3 expression. Pretreatment of CCA cells with genistein at 50 µM, but not NK-92 cells, significantly increased NK-92 cell killing ability over the untreated control, suggesting the ability of genistein to sensitize CCA cells. Interestingly, genistein treatment could greatly lower the expression of cFLIP, an anti-apoptotic protein involved in the immune escape pathway, in addition to upregulation of death receptors, Fas- and TRAIL-receptors, in CCA cells, which might be the underlying molecular mechanism of genistein to sensitize CCA to be susceptible to NK-92 cells. Taken together, this finding revealed the benefit of genistein as a sensitizer to enhance the efficiency of NK cell immunotherapy for CCA.

## 1. Introduction

Cholangiocarcinoma (CCA) is a lethal malignancy of the bile duct epithelium. The incidence of CCA is increasing in the past few decades worldwide [1]. The highest prevalence has been reported in Thailand, specifically in the Northeastern regions, where liver fluke, *Opisthorchis viverrini*, is an endemic [2]. Based on anatomical location, CCA is classified into three subtypes including intrahepatic (iCCA), perihilar (pCCA), and distal (dCCA), which have differences in epidemiology, prognosis, and treatment options [3]. Surgery is as only curative treatment, but it was estimated that only 35% of the patients with an early diagnosis were resectable [4]. However, the treatment outcome of CCA could not reach the satisfaction of prolonging survival rate because of cancer recurrence [5]. For the unresectable patients or those with advanced diseases, systemic therapies such as chemotherapy, radiotherapy, and targeted therapy have been used but with the limited effectiveness [6] because of the development of resistance to these therapies. Accordingly, the search for more effective treatment is required to decrease the mortality rate, prolong survival, and improve the quality of the patients’ life.

A new horizon of cancer therapy is immunotherapy to harness the patients’ immune system to fight against cancer by improving specificity and efficiency of anti-tumor capacity of immune cells [3]. Immunotherapies for CCA including immune checkpoint inhibitors (ICIs) [7], chimeric antigen receptor (CAR) T-cells [8], and tumor vaccines [9] have significantly been developed in the past few years [6,10]. However, the beneficial gains from balancing between efficacy and safety of immunotherapy in CCA is unclear and controversial [5]. One of the attractive innate immune cells, that plays an important role in tumor immunosurveillance, is natural killer (NK) cells. The NK cell immunotherapy has increasingly received attention because it could avoid the graft-versus-host disease (GVHD) [11]. The NK-92 cell line has been reported to be used for immunotherapy, which is not only providing strong cytotoxicity without GVHD concern as the primary NK cells but also their homogenous population and reduced time-consuming process for preparation, making NK-92 cells as an ideal for off-the-shelf therapy [12]. The data of multiple trial studies showed NK-92 cell infusion was well-tolerated without significant adverse clinical effects and achieved promising anti-cancer activities [13,14,15]. However, the efficacy and responsiveness of NK cell-based immunotherapies in CCA remain unclear [16]. Notably, activation of aberrant anti-apoptosis cascade in CCA is a mechanism to enhance resistance to NK cell-mediated apoptosis [17]. Overexpression of an anti-apoptotic protein, namely cellular FLICE (FADD-like IL-1β-converting enzyme)-inhibitory protein (cFLIP), in CCA cells has been reported to mediate resistance to cytotoxic immune cells through reduction of apoptotic mechanism and this might also promote immune evasion on CCA [18]. Thus, the strategy to improve NK cytotoxicity and suppress the immune escapes mechanism in CCA is an interesting therapeutic approach.

Chemotherapeutic drugs and natural compounds have been reported to be able to sensitize cancer cells by reversal of resistant cancer cells to be susceptible to cytotoxic immune cells [19,20,21,22]. Compared with traditional chemotherapies, natural compounds showed strong multitargeted anti-cancer effects and contain less side effects [23]. Among the phytochemical substances that were widely used as a sensitizing agent, genistein is one of the promising chemosensitizers for the chemotherapy [24]. Genistein, an isoflavone derived from soybean products, was well documented for its anti-cancer effects mediating through various pathways, such as induction of apoptosis, inhibition of angiogenesis and metastasis, reduction of cell proliferation, and decrease of inflammation [25,26,27,28,29]. However, the effect of genistein on the sensitization of CCA cells to be susceptible to NK cells remains unclear.

In the present study, we investigated the effect of genistein on the sensitization of CCA cells for NK-92 cytotoxicity. Its effects on the modulation of apoptotic and anti-apoptotic proteins were examined. Furthermore, the mechanism underlying the effect of genistein on sensitization of CCA cells, involving the alteration of death receptor expression, was also studied. The results of this study would provide a better understanding on the molecular mechanism of CCA cell sensitization by genistein, to be susceptible to NK cytotoxicity, which would signify the use of genistein in combination of NK cell immunotherapy.

## 2. Materials and Methods

### 2.1. Cell Culture

CCA cell lines including KKU055 (JCRB1551), KKU100 (JCRB1568), and KKU213A (JCRB1557) cells were originated from the CCA patients residing in opisthorchiasis endemic areas in the Northeastern Thailand. These cell lines were established by the researchers at the Faculty of Medicine, Khon Kaen University, Khon Kaen, Thailand, and deposited at the Japanese Collection of Research Bioresources Cell Bank (JCRB Cell Bank), where we obtained for our studies. The CCA cells were cultured in Gibco Dulbecco’s Modified Eagle Medium: Nutrient Mixture F-12 (DMEM/F12) (Thermo Fisher Scientific, Waltham, MA, USA) with 10% fetal bovine serum (Thermo Fisher Scientific, Waltham, MA, USA) at 37 °C under a humidified 5% CO_2_. NK-92 cells obtained from ATCC (ATCC, Manassas, VA, USA) were cultured in Minimum Essential Medium Eagle-α Modification supplemented with 12.5% horse serum (Thermo Fisher Scientific, Waltham, MA, USA), 2 mM L-glutamine, 0.02 mM inositol, 0.1 mM 2-mercaptoethanol, 0.02 mM folic acid, 100–200 U/mL recombinant IL-2 (R & D system, Minneapolis, MN, USA) and 12.5% fetal bovine serum.

### 2.2. Cell Viability Assay

The cytotoxic effect of genistein on CCA cell lines was determined by cell viability assay. Briefly, KKU055, KKU100, and KKU213A cells were plated into a 96-well plate (10^4^ cells per well) a day before the experiment. Genistein (0–400 µM) was added to the cells and incubated for 24 and 48 h. At the indicated times, PrestoBlue^TM^ reagent (Invitrogen, Carlsbad, CA, USA) was added to the wells to stain viable cells, which maintained the reducing capacity. The changes of the reagent color reflecting the reducing capacity of the cells were measured at the absorbances of 560 and 590 nm by NS-100 NanoScan Microplate Reader (Hercuvan Lab System, Cambridge, UK). The percentage of cell viability was calculated, compared with that of the untreated control (set as 100%). The half-maximal cytotoxicity concentration (CC50) of each cell at each time point was analyzed by using non-linear regression in GraphPad Prism software, version 7.0a (GraphPad Software, Inc., San Diego, CA, USA).

In addition, cytotoxic effect of genistein on NK-92 cells was evaluated. NK-92 cells (10^5^ cells per well) were added into a 96-well plate in the presence of genistein at concentrations of 25, 50, and 100 µM for 24 and 48 h. Then, NK-92 cell viability was measured by PrestoBlue^TM^ reagent as previously described. The percentage of cell viability of NK-92 cells after being treated with genistein were compared with those of untreated and diluent-treated groups.

### 2.3. Flow Cytometry

To investigate the effect of genistein on inducing apoptotic cell death, annexin V/propidium iodide (PI) staining and the flow cytometry were performed. Briefly, KKU055, KKU100, and KKU213A (10^5^ cells per well) were plated in a 12-well plate and treated with genistein at a concentration of 25, 50, and 100 µM for 24 h. The cells were collected and washed with 50 µL of 1x binding buffer before adding 50 µL of buffer containing annexin V-APC and PI-PE (1:100 dilution) (Immunotools, Friesoythe, Germany). The cells were incubated at room temperature in a dark place for 15 min and apoptotic cells were detected by BD Accuri C6 Flow Cytometer (BD Biosciences, San Joes, CA, USA).

The effect of genistein on modulating the death receptors, Fas and TRAIL receptors, was determined in CCA cells. Briefly, genistein at the indicated concentrations was added into a 12-well plate containing KKU055, KKU100, and KKU213A cells (10^5^ cells per well) for 24 h. The cells were harvested and stained with anti-CD95-APC (cat. no. 17-0959-42; Invitrogen, Thermo Fisher Scientific, Waltham, MA, USA), anti-DR4-PE (cat. no. 12-6644-42; Invitrogen, Thermo Fisher Scientific, Waltham, MA, USA), or anti-DR5-PE (cat. no. 12-9908-42; Invitrogen, Thermo Fisher Scientific, Waltham, MA, USA) at dilution of 1:50. The abundances of death receptors including Fas receptor (CD95) and TRAIL receptors (DR4 and DR5) were detected by BD Accuri C6 Flow Cytometer (BD Biosciences, San Joes, CA, USA).

### 2.4. Immunoblotting Assay

Genistein at concentrations of 25, 50, and 100 µM was treated into three CCA cell lines (10^5^ cells per well in a 12-well plate) for 24 h. Treated cells were collected and lysed with lysis buffer (0.1% of NP-40 in PBS). Cell debris was removed by centrifugation at 8000 rpm for 1 min. The cell lysate was collected and subjected to protein separation by sodium dodecyl sulfate-polyacrylamide gel electrophoresis (SDS-PAGE). The protein was then blotted onto a nitrocellulose membrane and blocked with 3% skimmed milk in Tris-buffered saline containing 0.1% Tween-20 (0.1% TBST) for an hour. The following antibodies were used to detect the caspases and anti-apoptotic proteins, including mouse anti-procaspase 3, rabbit anti-procaspase 8, mouse anti-procaspase 9, rabbit anti-Bcl-2, rabbit anti-cFLIP, and rabbit anti-GAPDH (ABclonal, Woburn, MA, USA) at dilution of 1:1000. The membrane was washed three times with 0.1% TBST and secondary antibodies including goat anti-mouse and goat anti-rabbit antibodies (ABclonal, Woburn, MA, USA) at dilution of 1:1000 were subsequently used. The membrane was washed three times with 0.1% TBST before adding SuperSignal West Pico PLUS Chemiluminescent Substrate (Thermo Fisher Scientific, Waltham, MA, USA). The abundances of interested proteins were detected by the ImageQuant LAS 500 Chemiluminescent Imaging System (GE, Boston, MA, USA). The protein band intensity was analyzed by ImageJ software [30].

### 2.5. Killing Assay

**Figure 1:**
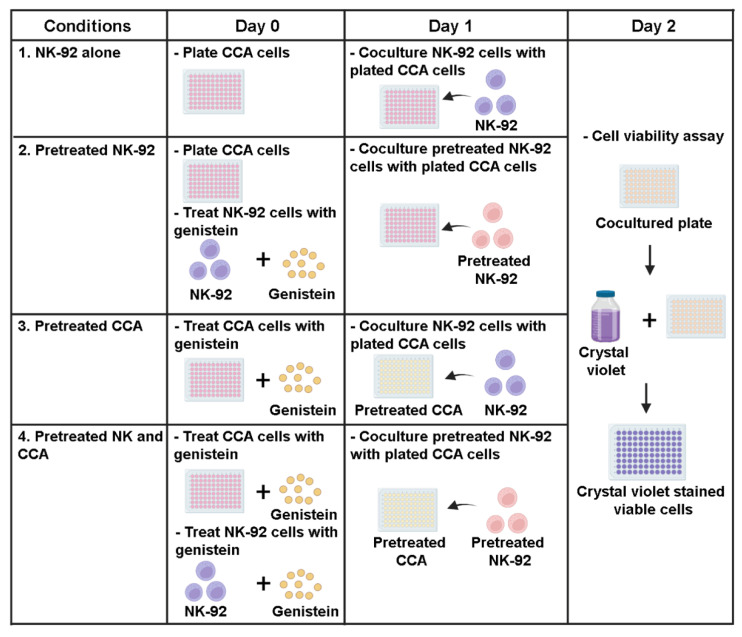
Sensitization effects of genistein on CCA cells to be killed by NK-92 cells in killing assay. The experiments were divided into 4 conditions: (1) NK-92 alone condition, CCA cells (target cells) and NK-92 cells (effector cells) were cocultured without the addition of genistein (control group), (2) Pretreated NK-92 condition, NK-92 cells were treated with 50 µM of genistein for 24 h prior to coculture with CCA cell lines, (3) Pretreated CCA condition, CCA cells were treated with 50 µM of genistein for 24 h prior to coculture with NK cells. (4) Combination of pretreated NK and CCA condition, NK cells and CCA cell lines were treated with 50 µM of genistein for 24 h and used in killing assay. In the killing assay, the E:T ratio was 1:1. The viable cells were measured after 24 h of coculture.

To investigate the effect of genistein on modulating NK-92 killing ability, the killing assay was performed. Briefly, KKU055, KKU100, and KKU213A cells were plated into a 96-well plate (10^4^ cells per well) a day before the experiment. The treatments were divided into 4 conditions (Figure 1) including: (1) NK-92 alone, (2) pretreated NK-92, (3) pretreated CCA cells, and (4) combination of pretreated NK and CCA. In the NK-92 alone condition: the procedure was performed by cocultured CCA cells (target cells) with NK-92 cells (effector cells) without the addition of genistein and this condition was set as a control group. In the pretreated NK-92 condition: genistein at 50 µM was used to treat NK-92 cells for 24 h prior to coculturing with CCA cell lines. In the pretreated CCA condition: genistein at 50 µM was used to treat CCA cells for 24 h prior to coculturing with NK-92 cells. In the combination of pretreated NK and CCA condition: both NK-92 and CCA cells were treated with 50 µM genistein as described and subjected to the killing assay. After coculturing for 24 h with an E:T ratio of 1:1, a culture medium containing NK-92 cells and death target cells was removed. The living cells attached to the plate were then determined by crystal violet staining. The stained cells were solubilized by using absolute methanol and absorbance at 570 nm, representing the number of living cells, were measured. The percentage of cell death relative to untreated control (no NK and genistein) was calculated by using the following equation:

Cell death (%) = 100 − [(OD570 treated cell/OD570 untreated cell) × 100]

### 2.6. Statistical Analysis

One-way ANOVA followed by Tukey’s post-hoc of GraphPad Prism software, version 7.0a (GraphPad Software, Inc., San Diego, CA, USA) was generally used for statistical analysis in each experiment. Three independent experiments were performed and illustrated as mean ± standard error of the mean (SEM).

## 3. Results

### 3.1. Genistein Induced Cell Death in CCA Cell Lines

**Figure 2:**
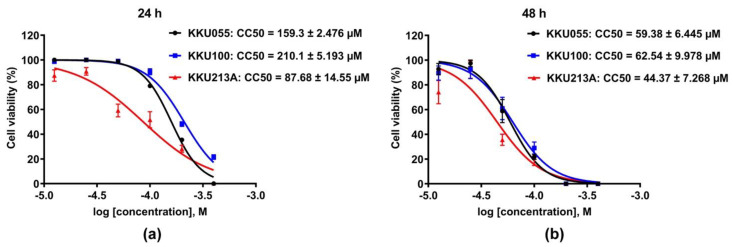
Cytotoxic effect of genistein on CCA cell lines. The percentage of cell viability of KKU055, KKU100, and KKU213A cells after genistein treatment at the concentrations varied from 0–400 µM were determined at 24 (**a**) and 48 (**b**) hours. The percentage of cell viability at the log of molar concentration was used to calculate CC50 value by using non-linear regression fitted with log inhibitor vs. normalized response (variable slope) of GraphPad Prism software, version 7.0a.

The cytotoxic effects of genistein on three different CCA cell lines, KKU055, KKU100, and KKU213A cells, were evaluated and compared by using a cell viability assay. The result showed that genistein greatly caused cell death in all CCA cell lines (Figure 2). KKU213A cells were the most sensitive to genistein with CC50, accessed at 24 and 48 h of 87.68 ± 14.55 and 44.37 ± 7.268 µM followed by KKU055 cells with CC50 of 159.3 ± 2.476 and 59.38 ± 6.445 µM, and KKU100 cells, the most resistant to genistein, with CC50 of 210.1 ± 5.193 and 62.54 ± 9.978 µM, respectively (Figure 2a,b).

### 3.2. Genistein Activated Extrinsic Apoptotic Pathway in CCA Cell Lines

**Figure 3:**
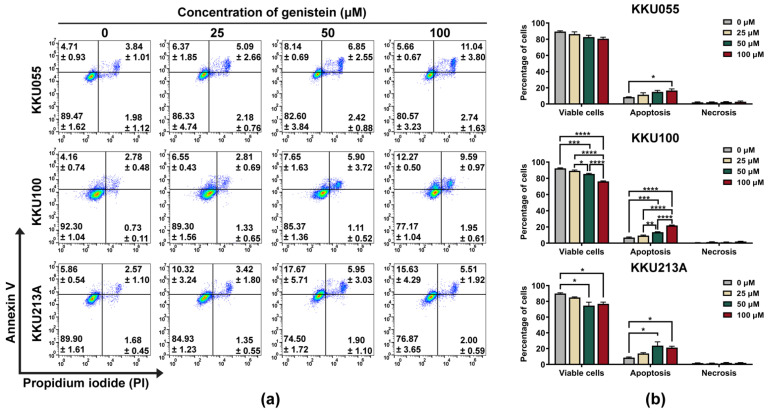
Genistein induced apoptosis cell death in CCA cell lines. The apoptosis cell death was detected by using annexin V/PI staining and flow cytometry. The dot plot showed the population of KKU055, KKU100, and KKU213A cells after 24-h treatment of genistein at the concentrations of 25, 50, and 100 µM. The number of the cells in early apoptosis (upper-left), late apoptosis (upper-right), and necrosis (bottom-right) were shown in mean ± SEM value (**a**). The percentage of the cells in each stage including viable cells, apoptosis (early and late stage), or necrosis was calculated relative to that of non-treatment control (set as 100%). The statistically differences were compared with diluent control group (* indicates *p* < 0.05, ** indicates *p* < 0.01, *** indicates *p* < 0.001, and **** indicates *p* < 0.0001) (**b**).

We further investigated the pathway of cell death induced by genistein by using annexin V/PI staining and examination of procaspase-3, procaspase-8, and procaspase-9 expression by immunoblotting analysis. By annexin V/PI staining and flow cytometry analysis, genistein treatment caused the apoptosis in KKU055, KKU100, and KKU213A cells which mostly turned CCA cells into late apoptosis (annexin V and PI doubled positive) in a dose-dependent manner (Figure 3a). Similar to the results of the cell viability assay, the percentage of total apoptosis was mostly observed in KKU213A cells followed by KKU055 and KKU100 cells, which were 23.62 ± 4.873, 14.99 ± 1.794, 13.55 ± 0.545 respectively (Figure 3b).

**Figure 4:**
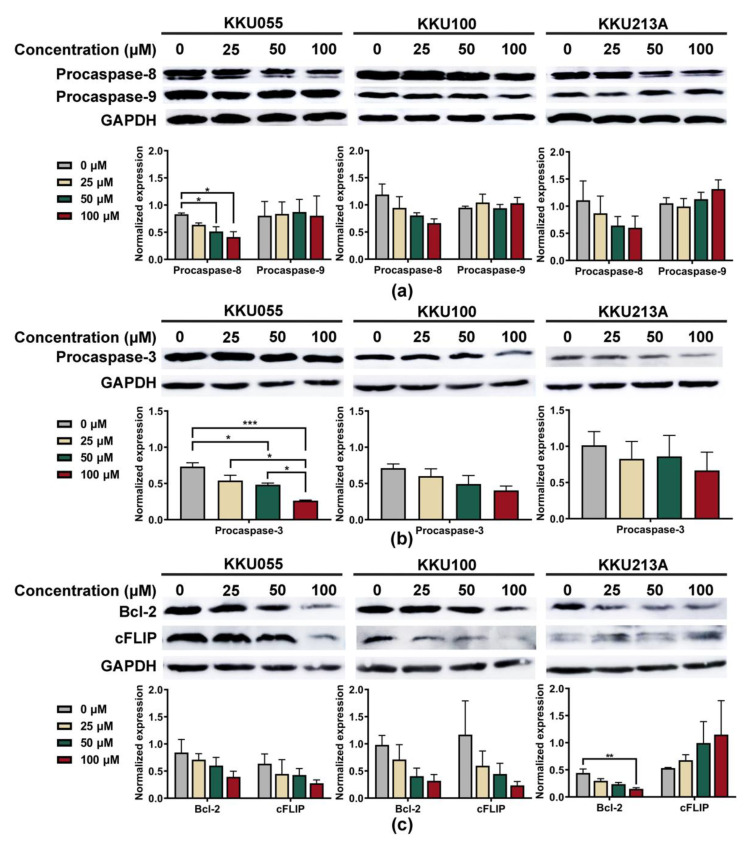
Genistein induced apoptosis through an extrinsic pathway in CCA cell lines. The immunoblotting technique was used to examine the alterations of apoptotic marker proteins, including (**a**) procaspase-8 and procaspase-9, and (**b**) procaspase-3 after 24-h treatment of genistein at the concentration of 25, 50, and 100 µM in KKU055, KKU100, and KKU213A cells. The changes in anti-apoptotic proteins: (**c**) Bcl-2 and cFLIP proteins, were also detected. Band intensity representing protein quantity was normalized with that of GAPDH. The statistical differences were tested by comparing to the value of diluent control (* indicates *p* < 0.05, ** indicates *p* < 0.01, and *** indicates *p* < 0.001).

To explore apoptosis pathway, we investigated alterations of procaspase-3, procaspase-8, and procaspase-9 expression to clarify whether genistein could promote extrinsic or intrinsic apoptosis pathways. The expression levels were normalized to that of GAPDH and compared among CCA cell lines. Genistein treatment caused the reduction of procaspase-8 but not procaspase-9 in a dose-dependent manner (Figure 4a). At the highest tested concentration of genistein (100 µM), procaspase-8 expressions of KKU055, KKU100, and KKU213A cells were decreased from 0.831 ± 0.024, 1.191 ± 0.193, and 0.867 ± 0.279 to 0.413 ± 0.097, 0.664 ± 0.135, and 0.262 ± 0.091, when compared with diluent control, respectively (Figure 4a). The reduction of procaspase-3 was determined to indicate the activation of the apoptotic cascade which finally resulted in the formation of apoptotic bodies [31]. As expected, genistein could significantly cause the reduction of procaspase-3 expression, especially in the KKU055 cells, in which the concentration of 50 µM of genistein was able to decrease procaspase-3 expression from 0.734 ± 0.052 to 0.483 ± 0.022 (Figure 4b).

We further investigated the alteration of anti-apoptotic proteins, including Bcl-2 and cFLIP, which play a role in controlling the survival of the cancer cells. The result showed that genistein at 100 µM significantly reduced the expression level of Bcl-2 protein in KKU213A cells from 0.443 ± 0.073 to 0.149 ± 0.022 (Figure 4c). Furthermore, the lowering of cFLIP protein was observed after genistein treatment and could lower the expression from 0.636 ± 0.180 and 1.169 ± 0.621 to 0.276 ± 0.061 and 0.235 ± 0.071 in KKU055 and KKU100 cells, respectively, but not in KKU213A cells (Figure 4c).

### 3.3. Genistein Enhanced Sensitivity of CCA Cell Lines to NK-92 Cell Killing

Expressions of anti-apoptotic proteins have been reported to be involved with the immune escape mechanism of cancer [32]. Since genistein could lower expressions of the anti-apoptotic proteins, we asked whether it could improve the killing ability of immune cells. Thus, the effect of genistein on enhancement of NK-92 killing against CCA cell lines was examined by comparing 4 experimental conditions including: (1) NK-92 alone, (2) pretreated NK-92, (3) pretreated CCA cells, and (4) combination of pretreated-NK and CCA cells (Figure 1).

**Figure 5:**
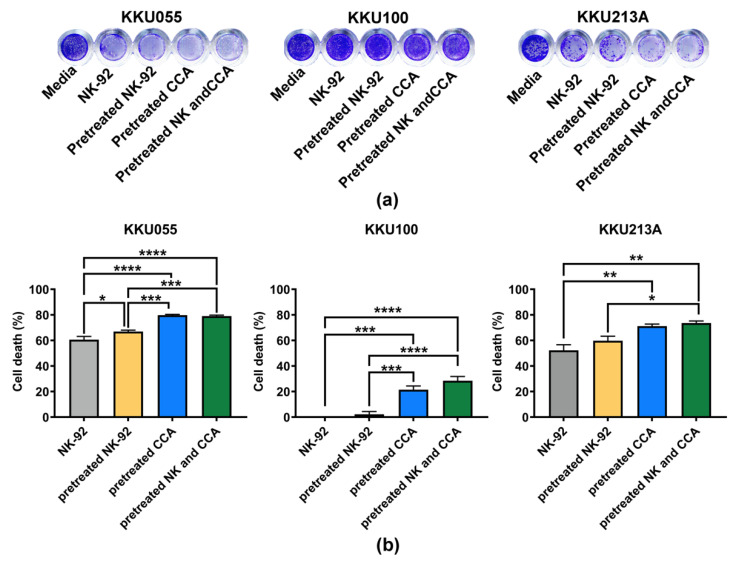
Killing of CCA cells by NK-92 cells examined by killing assay. (**a**) The living cells were determined after 24 h of coculturing CCA cells and NK-92 cells by crystal violet staining by using 4 experimental conditions including (1) NK-92 alone, (2) pretreated NK-92, (3) pretreated CCA, and (4) combination of pretreated NK and pretreated CCA condition. (**b**) The percentages of cell death (% cell death), represented in bar graph, were tested for statistical differences between each condition and the control group (NK-92 alone) (* indicates *p* < 0.05, ** indicates *p* < 0.01, *** indicates *p* < 0.001, and **** indicates *p* < 0.0001).

We examined the effect of genistein on NK-92 cell viability and found that after treatment with genistein at the 25, 50, and 100 µM, more than 85% of NK-92 cells were viable (Supplementary Figure S1), suggesting no or minimal effect of genistein on NK-92 cell viability at these tested concentrations. The coculture of CCA and NK-92 cells was performed at an E:T ratio of 1:1 for 24 h. The living cells, which remained attached to the plate, were stained with crystal violet (Figure 5a). The result showed that original NK-92 cells had a great ability to kill KKU055 and KKU213A cells but not KKU100 cells. They could kill approximately 60.65 ± 2.48% and 52.25 ± 4.34% of KKU055 and KKU213A cells, respectively. In the pretreated NK-92 condition, cell deaths were slightly increased to 66.94 ± 1.13% for KKU055 cells and 59.80 ± 3.48% for KKU213A cells whereas no change was observed for KKU100 cells (Figure 5b). Interestingly, in pretreated CCA conditions, genistein could significantly improve the NK killing by increasing percentage of cell deaths up to 79.83 ± 0.56% for KKU055 cells, 21.43 ± 2.91% for KKU100 cells, and 71.18 ± 1.64 for KKU213A cells. In the combination of pretreated NK-92 cells and pretreated CCA cells, percentage of cell death were increased to similar levels as those of the pretreated CCA alone (79.07 ± 0.76% for KKU055 cells, 28.47 ± 3.35% for KKU100 cells, and 73.59 ± 1.62% for KKU213A cells).

### 3.4. Genistein Increased Expression of Death Receptors on CCA Cell Lines

**Figure 6:**
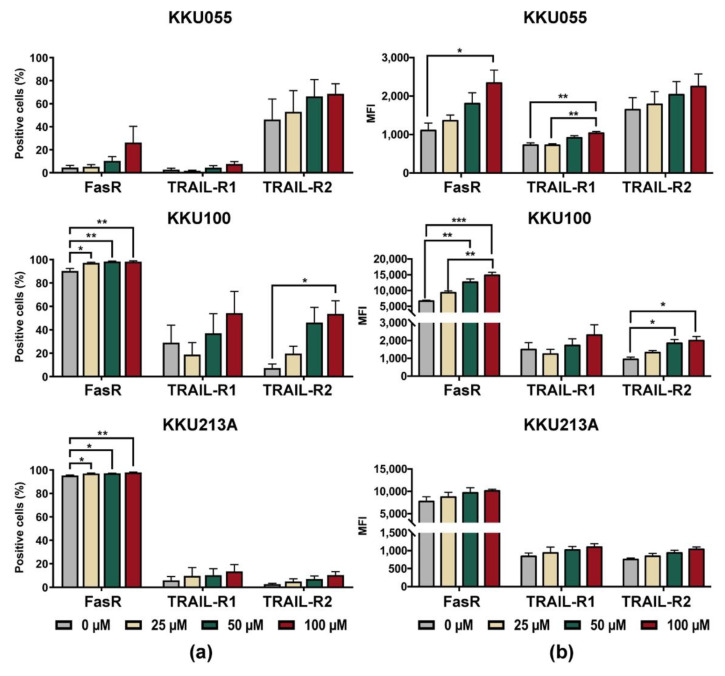
Changes of cell surface expression levels of death receptors after treatment of KKU055, KKU100, and KKU213A cells with genistein, analyzed by flow cytometry. The cell surface expression of FasR (CD95), TRAIL-R1 (DR4), and TRAIL-R2 (DR5) were analyzed after treatment of CCA cells with genistein for 24 h. (**a**) Percentage of positive cells, and (**b**) mean fluorescent intensities (MFI) were quantified, compared to that of the diluent control. The statistical analyses were performed to compare between the results of treated condition and that of the diluent control (* indicates *p* < 0.05, ** indicates *p* < 0.01, and *** indicates *p* < 0.001).

Based on the result that genistein could improve killing activity of NK-92 cells in the pretreated CCA condition, alterations of death receptors on CCA cells were, thus, investigated. The changes of expression levels of Fas receptor (FasR/CD95) and TRAIL receptors, including TRAIL-R1 (DR4) and TRAIL-R2 (DR5), were determined on the cell surface of CCA cell lines after treatment with genistein at concentrations of 25, 50, and 100 µM (Supplementary Figure S2 and Figure 6). The results showed that genistein treatment increased proportions of FasR positive cells (Figure 6a) in corresponding to the mean fluorescence intensity (MFI) of FasR which increased in a dose-dependent manner (Figure 6b). Genistein at 100 µM could augment the FasR positive cells up to 98.3 ± 0.67% for KKU100 cells, 97.97 ± 0.35% for KKU213A cells, and 26.26 ± 14.17% for KKU055 cells (Figure 6b).

In the analyses of positive cells, TRAIL-R1 (DR4) positive cells were greatly increased in KKU100 cells but fewer in KKU213A and KKU055 cells. Treatment of CCA cells with genistein at 100 µM increased the TRAIL-R1 positive cells up to 54.3 ± 18.52%, 13.66 ± 5.67%, and 7.59 ± 2.16% in KKU100, KKU213A, and KKU055 cells, respectively, which were higher than those of untreated control (29.05 ± 14.89%, 5.96 ± 3.28%, and 2.69 ± 1.29%, respectively). The expression of TRAIL-R2 (DR5) was significantly increased in KKU100 cells, from 7.31 ± 3.50% to 53.63 ± 11.14% (Figure 6a). The changes in KKU055 and KKU213A cells were less pronounced, from 46.23 ± 17.82% to 68.6 ± 8.79% in KKU055 cells and 2.68 ± 7.30% to 10.42 ± 2.99% in KKU213A cells after treatment with genistein at 100 µM (Figure 6a).

In the analyses of mean fluorescence intensity (MFI), the expressions of all death receptors including FasR, TRAIL-R1, and TRAIL-R2 were increased after CCA cell lines were treated with genistein (Figure 6b). Treatment with 100 µM significantly increased FasR expression in KKU055 and KKU100 cells but caused only the slight effect in KKU213A cells. The changes in TRAIL-R expressions were less pronounced than that of FasR but their alterations were significant in the genistein-treated KKU055 and KKU100 cells.

## 4. Discussion

CCA is an aggressive type of cancer with poor prognosis and disease outcome [33]. It is highly resistant to the standard therapy, resulting in a very low survival rate (fewer than 12 months), especially for those with advanced disease, counted for 75% of the patients [34,35]. Thus, the development of a new approach is needed to fight against CCA that currently has increasing incidence. For decades, there is a huge attempt worldwide to discover a new therapeutic approach for CCA, including the development of immunotherapy; however, the efficacy of this new treatment versus its safety remains to be investigated.

The high genetic heterogeneity and desmoplastic stroma of CCA may provide a strong microenvironment that has negative impact to the treatment, such as chemotherapeutic drug delivery to the tumor, promotion of tumor cell proliferation, prevention of the tumor from immune surveillance and the induction of apoptosis [5,36]. Furthermore, the aberrantly activated apoptosis pathway in CCA limited efficiency of immunotherapy in this cancer type since it has been reported to suppress immune cell function and promote immune escapes mechanism [18]. Therefore, inhibition of anti-apoptotic signaling in CCA cells, may serve as a feasible approach to enhance anti-cancer activity of immune cells. In the present study, we have demonstrated the effect of genistein on enhancing sensitivity of CCA cell lines to NK-92 cell-mediated cytotoxicity.

The anti-cancer activity of genistein was studied in three different CCA cell lines, which were originally established in Thai CCA patients. Generally, these three cell lines, including KKU055, KKU100, and KKU213A cells, contain different characteristics in which KKU055 and KKU100 cells were categorized into poorly differentiated cholangiocarcinoma cells whereas KKU213A cells were grouped into well-differentiated CCA cell line [37,38]. Comparing these three cell lines in our study, KKU213A cells showed the most sensitivity to genistein with the CC50 accessed at 24 and 48 h of 87.68 and 44.37 µM, followed by KKU055 cells (Figure 2). The high resistance was observed in KKU100 cells with the CC50 value of 210.1 µM judged at 24 h of treatment, which was 2.4-fold higher than that of KKU213A cells. The diversity of genetic background and the origin of each cell line itself might serve as the key factor resulting in the different drug sensitivity as it has been reported to contribute to drug metabolism and drug responsiveness [39,40]. KKU055 cells were usually sensitive to the standard chemotherapeutic agents [41]. KKU100 cells exhibited resistance trait to 5-FU, and etoposide, but were sensitive to doxorubicin while KKU213A cells was the more sensitive to gemcitabine than KKU100 cells [41,42]. In line with the results studied by others, our result revealed the impact of cancer heterogeneity on the varied responses to genistein treatment, which suggests the benefit of drug response assessments and the point of the requirement of biological markers for prediction of drug response and evaluation of disease outcome.

The apoptosis induction of genistein was predominantly observed in CCA cell lines based on annexin V/PI staining (Figure 3). Genistein at a concentration of 50 µM could trigger apoptosis in KKU055 and KKU213A cells through an extrinsic pathway, based on the reduction of procaspase-8 and procaspase-3 caused by caspase cascade activation and cleavage. However, there was a less significant change in apoptotic protein alteration in KKU100 cells (Figure 4a,b). The effect of genistein to promote cancer cell death has been reported in several cancers but its lethal doses depended on types of cancer cells, i.e., 50-µM genistein could trigger apoptosis via increased expression of caspase-3 in HT29 cells [43] whereas 100-µM genistein promoted apoptosis in MCF-7 cells, reflecting the difference of drug sensitivity among cancer cell types [44]. Interestingly, at the equal concentration to kill MCF-7 cells (100 µM), it did not promote cell death of CCD1059sK fibroblast cells (non-cancerous cell) [44], suggesting safety of genistein at this concentration or lower.

The underlying mechanism of genistein in promoting cancer cell death was through various pathways based on the multitargeted function of genistein. Genistein is well-documented to promote the cell cycle arrest via promoting G_2_/M cell cycle arrest, resulting in anti-proliferation effects in gastric [45], breast [46], and colon [28] carcinoma cells. Genistein was reported to promote cell death in intrahepatic CCA cell lines, HuCCA-1 and RNCCA-1, via inhibiting survival signaling of epidermal growth factor receptor (EGFR), AKT, and the mitogen-activated protein kinase (MAPK) cascade [47]. Alternatively, genistein caused alterations of anti-apoptotic proteins, including Bcl-2 and cFLIP, resulting in disruption of survival signaling, cancer proliferation, and induction of cancer cell apoptosis [25]. Bcl-2 and cFLIP play pivotal roles in the cancer resistance [44] and reduction of Bcl-2 and cFLIP participate in enhancing drug sensitivity. In CCA, treatment of BI6727 in KMCH-1 and Mz-Ch-1 caused the degradation of Bcl-2, which sensitized these CCA cell lines to cisplatin via apoptosis pathway [48]. The other protein, cFLIP, which is highly expressed in human intrahepatic CCA (iCCA) was reported to inhibit apoptosis and promote cancer proliferation [18]. In our study, genistein treatment showed to greatly reduce the expression of Bcl-2 in all three cell lines whereas cFLIP tended to be decreased in KKU055 and KKU100 cells in a dose-dependent fashion but not in KKU213A cells (Figure 4c), which might lead to the apoptosis of these two CCA cells.

Alteration of cFLIP protein can affect the endoplasmic reticulum (ER) stress-inducing apoptosis pathway [49]. ER stress-induced apoptosis via the activation of caspase-12, which can subsequently activate caspase-9 and caspase-3 to induce apoptosis [49]. Higher expression of cFLIP protein remarkedly inhibited ER stress in colon cancer cells and its downregulation by RNA interference greatly caused ER-stress-induced apoptosis in either 2D culture or multicellular tumor spheroid [50]. Interestingly, cFLIP downregulation in colon cancer cells showed to upregulate TRAIL-R2 expression, suggesting the possibility of cFLIP on the activation of TRAIL death pathway [50]. Moreover, the involvement of cFLIP in the resistance of Fas-mediated apoptosis has been reported in several cancers, including CCA [18,51,52]. Treatment of Fas agonist failed to promote cancer cell death in highly expressed cFLIP lymphoma (Karpas 299 and SU-DHL1), which could be sensitized by using cFLIP RNA interference [53]. In CCA, the overexpression of cFLIP has been reported in human iCCA specimens, compared to that in the normal duct [18]. Interestingly, cFLIP expression was increased upon coculture with PBMC which might contribute to hindering apoptosis of iCCA cells, resulting in cancer proliferation and immune escape mechanism [18].

Remarkably, TRAIL and Fas signaling cascades are associated with anti-tumor activity of the immune cells. Activated NK cells mediated anti-tumor activity mainly through releasing cytolytic granules (perforin and granzymes), which could promote either the caspase-dependent or independent apoptosis of cancer cells [54]. On the other hand, NK cells can switch their killing mechanism to trigger the TRAIL- and Fas-death receptor pathway and activate the subsequent signaling cascade of apoptosis [55]. Apart from the killing ability, the activation of NK cell function could promote the releasing of chemokines and cytokines, which can modulate adaptive immune response [56]. According to the potential role of cFLIP on TRAIL- or Fas-mediated cell death and our result that genistein could reduce the expression level of cFLIP, we thus investigated the immunosensitization effect of genistein to enhance NK killing activity and its association to TRAIL- and Fas-death receptor pathway. Comparing four different conditions performed in our study, the most potential condition was the pretreatment of genistein in CCA prior to coculture with NK cells (Figure 5). Pretreatment of NK-92 cells could not significantly improve the NK-92 cell killing ability and the result of combination of pretreated CCA cells and pretreated NK-92 cells was not significantly different from those of pretreated CCA alone, suggesting the effect of genistein mainly through the effect of genistein to CCA cells (Figure 5). The improvement could be observed in all CCA cell lines, which increased by approximately 20% of cell death (Figure 5). The association to TRAIL- and Fas-death receptor pathway was investigated by determining TRAIL- and Fas-death receptor expression, which revealed the upregulation of FasR (CD95), TRAIL-R1 (DR4), and TRAIL-R2 (DR5) after treatment with genistein in a dose-dependent manner (Figure 6).

Several studies reported the involvement of the TRAIL- and Fas-death receptor in enhancing the NK activity [57,58,59,60]. The proteasome inhibitor, bortezomib, was demonstrated to modulate FasR and TRAIL-R2 (DR5) in breast cancer [57] and leukemia [58]. Interestingly, this improvement of NK killing activity was found in perforin-deficient NK cells and could be inhibited by neutralizing antibodies to FasR and TRAIL-R, suggesting the NK cell-mediated cytotoxicity was promoted through TRAIL- and Fas-death receptor pathway [59]. Previous studies from our group, the increase of TRAIL-R after cordycepin treatment [22] and FasR after doxorubicin [21] associated with improvement of NK-92 cell killing activity in CCA and breast cancer, respectively, which emphasized the potential of FasR and TRAIL-R2 (DR5) modulation on NK cell killing activity. In this study, we demonstrated the effect of genistein on increasing either FasR or TRIAL-R expression in which the change of FasR expression was predominated in three cell lines over that of TRAIL-R (Figure 6). These changes could be the mechanism to promote genistein-treated CCA cells to be more sensitive to NK-92 cytotoxicity.

Compared with the chemotherapeutic drugs which have been reported to sensitize the cancer cells to the immune cells such as bortezomib [60] or doxorubicin [21], the effective dose (50–100 µM) of genistein was much higher than that of bortezomib or doxorubicin which in the nanomolar range. However, genistein is preferable in terms of safety which could avoid the serious effect of chemotherapeutic drugs. As such in addition to its anti-cancer effect, genistein could be used in long-term treatment to directly control the tumor growth/progression and enhance the NK killing ability. Recently, our group reported the immunosensitization effect of cordycepin, a bioactive compound of some *Cordyceps* spp. [22]. At a comparable concentration to genistein, cordycepin greatly increased the TRAIL-R expression and significantly improved the NK function to kill KKU213A but not KKU055 and KKU100 [22]. Since the effect of cordycepin was through TRAIL-R cascade whereas genistein was mainly through FasR, those suggested the possibility of the combination treatment to achieve the synergistic effect. However, further investigation is needed to evaluate the efficiency and effective dose of either genistein or cordycepin in head-to-head comparison and to compare with combined genistein-cordycepin treatment.

Genistein has the advantage of safety. Genistein was recommended to use for prevention of postmenopausal bone loss [61]. The phase 1 clinical trials in 40 healthy volunteers revealed that genistein at the oral doses of 30, 60, 150, and 300 mg (molecular weight of genistein is 270.24 g/mol) was rapidly absorbed and no significant side effects on vital signs, ECG, and clinical laboratory parameters were observed [61]. However, the absorption of genistein seemed to be limited and reached the mean Cmax values of 1808.0 ng/mL after 4.0 to 6.0 h at 300 mg dose [61]. Notably, the route of administration has been reported previously as the most crucial factor in considering the effect of genistein since the absorption through the gastrointestinal tract could limit the bioavailability and biological activity of the oral administration [62]. The injection to bypass the limitation of absorption and metabolism should be considered as an alternative approach to gain a high dose of genistein, especially in the case of disease treatment such as cancer. However, it requires a different level of safety/toxicity assessment to judge the optimal treatment dose.

Taken together, our study provided extended information regarding the effect of genistein on increasing sensitivity of CCA cells to the cytotoxic function of NK-92 cells, in which this sensitivity related to the induction of FasR or TRIAL-R expression on CCA cells upon the genistein treatment. However, the effects on improvement of other immune cells, sensitization of cancer to other immune cells, and the safety of genistein in the CCA patients require further investigation either in vivo or in clinical trials to evaluate the real benefits of genistein in CCA treatment.

## Data Availability

Not applicable.

## Supplementary Materials

The following supporting information can be downloaded at: https://www.mdpi.com/article/10.3390/biology11081098/s1, Figure S1: Cell viability of NK cells after exposure with genistein for 24 and 48 h; Figure S2: Gating strategies KKU055, KKU100, and KKU213A.
