# Supplementary material for: Genistein Sensitizes Human Cholangiocarcinoma Cell Lines to Be Susceptible to Natural Killer Cells
Source: Biology (Basel). 2022 Jul 23;11(8):1098. doi: 10.3390/biology11081098 (PMC9330512; doi:10.3390/biology11081098)
Supplement: Supplementary file 1 [file biology-11-01098-s001.zip › biology-1819285-supplementary.pdf]

Supplementary Materials

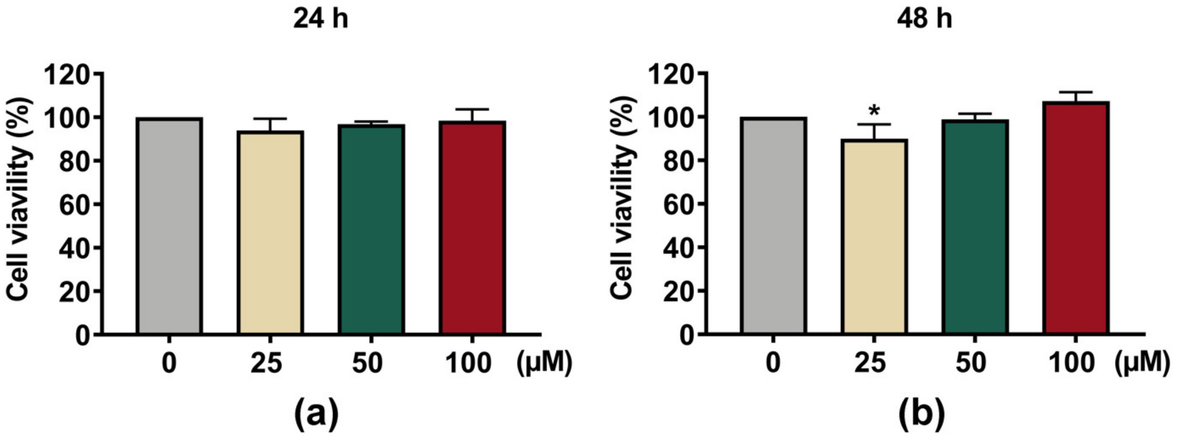

Figure S1. Cell viability of NK cells after exposed with genistein for 24 and 48 hours. After 24-hours of genistein treatment, genistein at concentration of 25, 50, and 100 μM slightly affected with cell viability of NK cells which were 93.92 ± 5.42%, 96.88 ± 1.21, and 98.42 ± 5.24, respectively. In addition of 48-hours treatment, the percentage of cell viability of NK cell were 89.97 ± 6.55%, 98.88 ± 2.59%, and 107.3 ± 4.05% after exposed with genistein at 25, 50, and 100 μM. The statistical tests (one-way ANOVA) were analyzed and compared with untreated group (0 μM) and * indicates $p < 0.05$. Moreover, the percentage of cell viability was exhibited in mean ± SD values (n = 3).

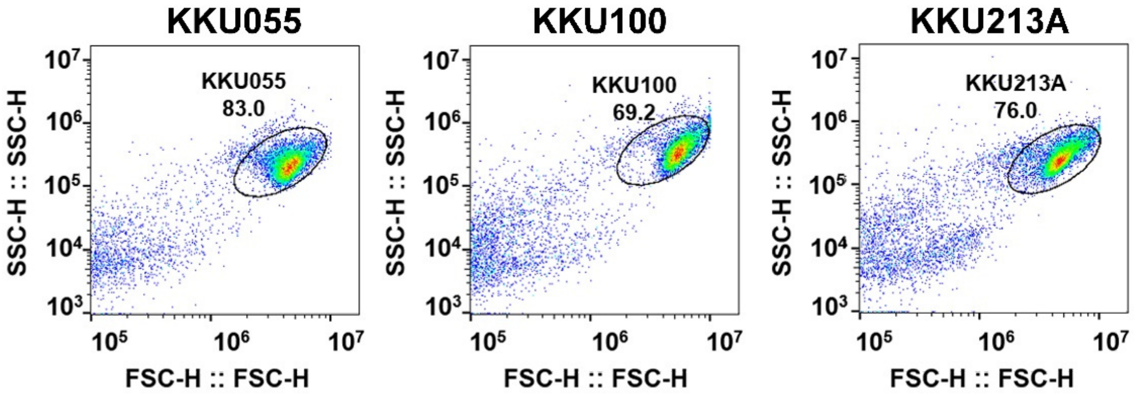

Figure S2. Gating strategies KKU055, KKU100, and KKU213A cells. The gating of CCA cell lines was performed to determine the changes on percentage of FasR and TRAIL-R (DR4 and DR5) positive cells after genistein treatment by using BD Accuri C6 Flow Cytometer.
